# Screening of *CYP1B1* Arg368His as predominant mutation in North Indian primary open angle glaucoma and juvenile onset glaucoma patients

**DOI:** 10.22099/mbrc.2018.30630.1344

**Published:** 2018-12

**Authors:** Avneet Kaur, Vanita Vanita, JaiRup Singh

**Affiliations:** 1 Department of Biotechnology, GGDSD College, Sector-32C, Chandigarh (UT) India-160030; 2 Department of Human Genetics, Guru Nanak Dev University, Amritsar 143005, India

**Keywords:** North Indian; POAG; p.Arg368His; CYP1B1

## Abstract

In India, mutations in Cytochrome P450 (*CYP1B1*) are a predominant cause of not only primary congenital glaucoma (PCG) but also involved in primary open angle glaucoma (POAG) and juvenile onset glaucoma (JOAG). After ethical clearance, 100 POAG patients, 30 primary angle closure glaucoma (PACG) patients and 130 ethnically matched controls were recruited in this study. Genomic DNA was isolated from the blood and screened for p.Arg368His mutation in CYP1B1 by polymerase chain reaction-restriction fragment length polymorphism (PCR-RFLP). On PCR-RFLP, 10/100 cases (10%) were found positive for Arg368His mutation. In North Indian POAG cases studied, p.Arg368His mutation was found only in heterozygous state. The frequency of p.Arg368His *CYP1B1* mutation in heterozygote state (10.0%) observed in our study in North Indian POAG patients is the highest in comparison to frequency observed in other ethnic groups from Southern and Eastern India.

## INTRODUCTION

The glaucomas represent a heterogeneous group of optic neuropathies with complex genetic basis characterized by progressive degeneration of retinal ganglion cells. It is characterized primarily by progressive degeneration of the optic nerve associated with visual field loss, with or without elevated intraocular pressure (IOP) [1]. The elevated IOP associated with glaucoma is due to increased resistance to the outflow of aqueous humor from the eye [2]. Glaucoma affects more than 70 million people worldwide with approximately 10% being bilaterally blind, making it the leading cause of irreversible blindness in the world [3]. In India, the estimated frequency of people who are blind due to glaucoma is 5 million [4]. In India, the number of people with glaucoma will increase to 16 million people in 2020 making India the second largest country after China to have the maximum number of glaucoma cases [5]. However, only 10-50% of people with glaucoma are aware of their glaucoma status [6]. Based on the age of onset and other clinical features, glaucoma has been classified into primary congenital glaucoma (PCG), juvenile-onset open-angle glaucoma (JOAG), and adult-onset primary open-angle glaucoma (POAG), primary angle closure glaucoma (PACG) [7].

The most common form of glaucoma is adult onset POAG that affects 50% of all the cases of glaucoma [7]. POAG is a multifactor optic neuropathy in which there is a characteristic acquired loss of optic nerve fibers, normal appearing anterior chamber angle, visual fields defects, and raised IOP. There are two forms of POAG: juvenile onset and adult onset. Usually, JOAG may manifest clinically between the ages of 3 and 30 years, while adult POAG manifests clinically after the age of 40 [8, 9]. Patients with JOAG usually have high intraocular pressure (IOP), advanced glaucomatous optic neuropathy and severe visual field defects [10].

Glaucoma is a genetically heterogeneous disease and linkage studies have shown the existence of 23 loci [11]. These loci include four genes: Myocilin/trabecular meshwork-induced glucocorticoid response protein (*MYOC*) (OMIM 601652) at 1q21-q31(12), Optineurin (*OPTN*) [13] at 10p14-p15, and WD repeat domain 36 WDR36) at 5q22.1 [14], *CYP1B1* at 2p22-p [15]. In Indian population, the MYOC has been shown to cause glaucoma in 2-4% of POAG cases [16, 17]. The cytochrome P450 (CYP1B1) has been shown to cause PCG, JOAG, and POAG in various population worldwide [18-20]. Various reports have reported the predominance of *CYP1B1* mutations in POAG and JOAG patients [20, 21-26]. The most common *CYP1B1* mutation in Indian POAG and JOAG patients are p.E229K and p.R368H [21, 27]. *CYP1B1* is the modifier locus for POAG that together with *MYOC* mutation expedite the progression from adult onset to a juvenile form in a digenic mode of inheritance [19]. However, *CYP1B1* alone can be responsible for JOAG in Indian as well as Western population [21, 28]. 

India has a heterogeneous population being ethno-linguistic different. The North Indian population is predominantly Aryan population while the Southern Indian population is Dravidian with totally morphological phenotype and genetic background. Since in Southern and eastern Indian population *CYP1B1* is involved in the pathogenesis of POAG, there was a need for screening of North Indian POAG patients for common mutations in *CYP1B1*. The direct costs, such as the cost of its diagnosis and treatment, and the indirect costs involved exert a heavy burden on both the patients and their families [29]. Therefore, genetic screening for predominant mutation in various ethnic groups has emerged as a method to identify individuals who are genetically susceptible to POAG, to facilitate early treatment and/or prevention. The present study aims at identifying p.Arg368His mutations in *CYP1B1* in North Indian POAG patients , and also to establish genotype/phenotype correlations. The data from this preliminary study might help to create a cost effective screening method for early detection of POAG patients.

## MATERIALS AND METHODS

100 POAG and 30 PACG glaucoma cases from different parts of India who visited Dr. Daljit Singh Eye Hospital Amritsar, Punjab were collected. This study was approved consistent with the provisions of the Declaration of Helsinki. After informed consent, clinical evaluation of study subjects was undertaken. 100 unrelated age matched control subjects from same ethnicity as patients in our study were recruited. Controls were chosen to match the ethnic, geographic, and linguistic background of the patients. They underwent complete ophthalmic examinations and were free from glaucoma other major eye diseases. IOP was measured and recorded at the time they were recruited, and previous IOP data were collected from their medical records. They had IOP<22 mm Hg and had no first-degree relative with glaucoma.

**Clinical Examination:** The clinical examinations of the glaucoma patients were done using standard ophthalmic procedures. Ocular examinations included slit lamp examination, measurement of IOP by application tonometry (Goldmann), refraction and best-corrected Snellen visual acuity anterior chamber angle evaluation by Gonioscopy (Goldmann 3-mirror gonioscope; Shaffer's grading) to check whether the iridocorneal angles are closed or open. The patients were then classified into open angle or angle closure glaucoma. The optic disc was evaluated by dilating the pupil with tropicanamide to check the cup to disc ratio (C/D ratio). The patient’s visual fields were assessed with a Humphrey’s automated perimetry.

**Criteria for diagnosis** The glaucoma patients collected for this study were diagnosed on the basis of atrophic optic disc or visual field damage with IOP>24 mm Hg, filtering surgery in the past, IOP>30 mm Hg and absence of optic disc damage. Inclusion criteria for POAG patients included elevated intraocular pressure of >2l mm Hg, open iridocorneal angles of the anterior chambers on gonioscopy along with significant cupping of optic disc which was confirmed by checking C/D ratios, typical visual field defects in automated perimetry test. Patients with secondary forms of glaucoma, such as exfoliation syndrome or a history of ocular trauma were excluded. JOAG was diagnosed as POAG occurring before the age of 40 years. Inclusion criteria for PACG patients included shallower angles, conjunctival injection and symptoms like ocular pain, nausea/vomiting and history of intermittent blurring of vision. 

**PCR-RFLP:** The genomic DNA was isolated from fresh or stored blood samples by the organic extraction with slight modifications [30, 31]. The mutation screening was carried out with a polymerase chain reaction (PCR)-based restriction fragment length polymorphism (RFLP) assay. For mutation analysis, exon 3 of *CYP1B1* were amplified by PCR using primers described previously [32]. 

## RESULTS AND DISCUSSION

The present study was conducted on 100 POAG (adult onset POAG=92, JOAG=8) and 30 PACG and 130 controls. The adult onset POAG and JOAG cases were pooled together to form one group, i.e., POAG, The clinical features of the patients recruited in this study are given in Table 1. The 100 POAG cases comprised of 73 sporadic cases and 27 cases with positive family history. In, PACG cases, 25 cases were of sporadic type while 5 had positive family history. 

**Table 1:** Detailed information about genetically analyzed glaucoma patients (n=130)

**Type of glaucoma**	**Adult onset POAG**	**JOAG**	**PACG**
Frequency (No. of cases)	61.5% (92)	5.0% (8)	20.0% (30)
Males/females	67/25	6/2	18/12
Mean age of onset (years)	52.5±12.0	27.9±16.2	47.1±10.8
Sporadic cases	68 (74.0%)	5 (62.5%)	25 (83.4%)
Familial cases	24 (26.0%)	3 (37.5%)	5 (16.6%)
Mean IOP (mm Hg)	30.1±11.6	32.0±11.1	31.5±12.7

p.Arg368His mutation was screened in 100 POAG and 30 PACG patients by PCR-RFLP analysis (Table 2). The p.Arg368His mutation was found in 10 POAG patients. In POAG cases, p.Arg368His mutation was found only in heterozygous state (10/100). None of the individual in the control group was positive for this mutation. The ophthalmologic findings in patients with Arg368His mutation are presented in Table 2.

In the present study, the median age of disease onset in POAG patients with p.Arg368His *CYP1B1* mutation was 46.9 years (range 32-70). Worldwide, in POAG cases with *CYP1B1* mutations, the most remarkable feature is comparatively early age of diagnosis (below 60 years of age) with variable median age of disease onset across population i.e., 23.6 years (range 8-36) in Canadian population as the study was carried on early-onset glaucoma patients [19]; 40 years (range 13-52) in French population, 57 years (range 17-62) in Indian population [21] and 59.9 years (range 48-77) in Spanish population [33]. 

**Table 2:** The detailed information of glaucoma cases with mutations in *CYP1B1* indicating their genotypes, phenotypes, age of onset, sex, ethinicity and geographical location. Clinical details like IOP (mm Hg), C/D ratio are given for right and left eye, respectively.

**Patient’s ID**	**State**	**Sex**	**Ethinicity**	**Age of onset (yrs)**	**Phenotype**	**IOP** **(RE, LE)**	**C/D ratio** **(RE, LE)**	**Visual acuity** **(RE, LE)**
GL-23	Punjab	M	Hindu	52	POAG	16,30	0.3,1.0	Nt
GL-30	Bihar	M	Hindu	70	POAG	50,42	1.0,0.3	NoLP6/24
GL-40	J&K	M	Muslim	50	POAG	17,34	1.0,0.6	Nt
GL-49	Punjab	M	Muslim	50	POAG	38,52	0.6,0.8	6/12,6/60
GL-61	Punjab	F	Sikh	37	POAG	27,24	0.5,0.3	6/18,6/12
GL-122	Punjab	F	Sikh	34	JOAG	18,19*	0.5,0.5	6/6, 6/60
GL-126	H.P	M	Hindu	37	POAG	33,20	0.4,0.2	6/6, 6/6
GL-139	Punjab	M	Sikh	38	POAG	32,45	Nt	6/8,no LP
GL-147	Punjab	M	Sikh	45	POAG	31,26	Wnl,1.0	6/6p,6/12
GL-167	Punjab	F	Sikh	65	POAG	36,48	0.9,0.8	6/60,6/60

POAG: adult onset primary open angle glaucoma, JOAG: juvenile onset open angle glaucoma; IOP: intraocular pressure; C/D ratio: cup to disc ratio, RE and LE: right eye and left eye, respectively.

The phenotypic variability associated with p.Arg368His mutation in *CYP1B1* could be due to various unknown modifying factors; genetic or environmental may modulate the development and clinical course of glaucoma. The p. Arg368His mutation occurs at a CpG dinucleotide within the J-Helix of the heme binding region of *CYP1B1*. The high frequency of p. Arg368His (10%) in POAG cases was higher than reported earlier in other studies. In POAG cases, p.Arg368His mutation has been reported in one sporadic JOAG case (0.5%) from Eastern India [21], 10 POAG patients from Southern India (3.98%) [34] and two familial JOAG cases (3.3%) from Canada [19], 7 JOAG cases from Northern India [26].

In the present study, an increased frequency of p.Arg368His in heterozygous state was observed in all the POAG cases studied (10%, n=100). Heterozygous *CYP1B1* mutations associated with POAG cases have previously been reported in various populations belonging to France (4.6%, n=236) [20], Canada (5%, n=60) [19], Spain (10.9%, n=82) [33] and India (4.0%, n=200) [21]. In JOAG patients from North India, Arg368His mutation was found in both heterozygous and homozygous state [26].

The frequency of p.Arg368His *CYP1B1* mutation in heterozygote state observed in our study in North Indian POAG patients is the highest in comparison to frequency observed in other populations and ethnic groups [19, 21, 26]. The patients belonged to ethnically different as well as geographically diverse groups, and so the possibility of founder effect is ruled out. We observed higher frequency of p.Arg368His mutation in *CYP1B1* in our cases than *MYOC* mutations or any other *CYP1B1* mutations in Indian population. Thus, p.Arg368His mutation in *CYP1B1* could be a significant risk factor for POAG patients in Indian population. However, the frequency of this mutation could vary with ethnicity and geographic location of the population studied. It is felt that large scale screening of this mutation using inexpensive PCR-RFLP analyses in different ethnic groups of India, would help in identifying individuals at risk and assist in medical management of the disease at early stages. Furthermore, screening of common mutations in *CYP1B1* and *MYOC* will be done in patients with heterozygous *CYP1B1* mutation.
